# Requirements and Operational Guidelines for Secure and Sustainable Digital Phenotyping: Design and Development Study

**DOI:** 10.2196/20996

**Published:** 2021-04-07

**Authors:** Raj R Jagesar, Jacob A Vorstman, Martien J Kas

**Affiliations:** 1 Groningen Institute for Evolutionary Life Sciences University of Groningen Groningen Netherlands; 2 Program in Genetics and Genome Biology Research Institute, The Hospital for Sick Children Toronto, ON Canada; 3 Department of Psychiatry University of Toronto Toronto, ON Canada

**Keywords:** digital phenotyping, mobile behavioral monitoring, passive behavioral monitoring, smartphone-based behavioral monitoring, research data management, psychoinformatics, mobile phone

## Abstract

**Background:**

Digital phenotyping, the measurement of human behavioral phenotypes using personal devices, is rapidly gaining popularity. Novel initiatives, ranging from software prototypes to user-ready research platforms, are innovating the field of biomedical research and health care apps. One example is the BEHAPP project, which offers a fully managed digital phenotyping platform as a service. The innovative potential of digital phenotyping strategies resides among others in their capacity to objectively capture measurable and quantitative components of human behavior, such as diurnal rhythm, movement patterns, and communication, in a real-world setting. The rapid development of this field underscores the importance of reliability and safety of the platforms on which these novel tools are operated. Large-scale studies and regulated research spaces (eg, the pharmaceutical industry) have strict requirements for the software-based solutions they use. Security and sustainability are key to ensuring continuity and trust. However, the majority of behavioral monitoring initiatives have not originated primarily in these regulated research spaces, which may be why these components have been somewhat overlooked, impeding the further development and implementation of such platforms in a secure and sustainable way.

**Objective:**

This study aims to provide a primer on the requirements and operational guidelines for the development and operation of a secure behavioral monitoring platform.

**Methods:**

We draw from disciplines such as privacy law, information, and computer science to identify a set of requirements and operational guidelines focused on security and sustainability. Taken together, the requirements and guidelines form the foundation of the design and implementation of the BEHAPP behavioral monitoring platform.

**Results:**

We present the base BEHAPP data collection and analysis flow and explain how the various concepts from security and sustainability are addressed in the design.

**Conclusions:**

Digital phenotyping initiatives are steadily maturing. This study helps the field and surrounding stakeholders to reflect upon and progress toward secure and sustainable operation of digital phenotyping–driven research.

## Introduction

### Background

Digital phenotyping is the practice of collecting and analyzing objective, longitudinal, and possibly high-resolution data streams from personal devices, such as smartphones and wearables, which are descriptive of a person’s real life and real-time behavior [1]. Digital phenotyping provides a new and much more detailed perspective on human behavior, with the potential to innovate both research and clinical outcome measures in the health care space.

The field is currently still experimental, featuring many studies reporting on methodologies and pilot data, but it has yet to deliver replicable findings that may prove useful for clinical translation [2]. At present, much of the current efforts concentrate on correlating smartphone-derived data with clinical diagnoses and symptoms domains, whereas the next steps will examine to what extent such data can be exploited to bring about real positive changes in clinical care [3]. In addition, the ethical aspects of digital phenotyping are examined [4]. At any rate, the pace of innovation is fast, the field is expanding rapidly, and the sample size of studies is steadily increasing, raising the question of whether the applications and services at the backend of such programs are up to the task of continuing to support the operations. More concretely, participating in large cohort studies and operating in regulated research spaces require us to ensure that our platforms are sustainable (ie, maintainable and scalable [5]) and, most importantly, secure [6]. Human research invariably requires the participation of individuals willing to participate in studies after obtaining appropriate informed consent. Establishing trust and continuity is key, given the sensitivity of the data that we collect and the context in which we operate [7].

The current state of the art shows that security and sustainability are not at the forefront of design and execution of digital phenotyping initiatives, despite some awareness of security and privacy implications [8] and the high cost of sustainability [9] in the currently available tools. However, the uptake in the field dictates a necessary shift from the implementation of a limited set of security measures to a more mature and holistic conception of all aspects of sustainability and security as integral parts of software engineering, starting at the very first stages of design. In many current reports on behavioral monitoring platforms, these factors are only discussed in a limited fashion [10-12]. We do not want to suggest that these initiatives are necessarily insufficient in this respect, but in general, there appears to be a focus on the scope of phenotypic outcomes captured by the platform, whereas attention to aspects related to security and sustainability is relatively peripheral.

To address this gap, we present here a detailed description of the requirements and guidelines to responsibly develop and operate a behavioral monitoring platform, with specific consideration of the aforementioned concerns.

### About BEHAPP

**Figure 1:**
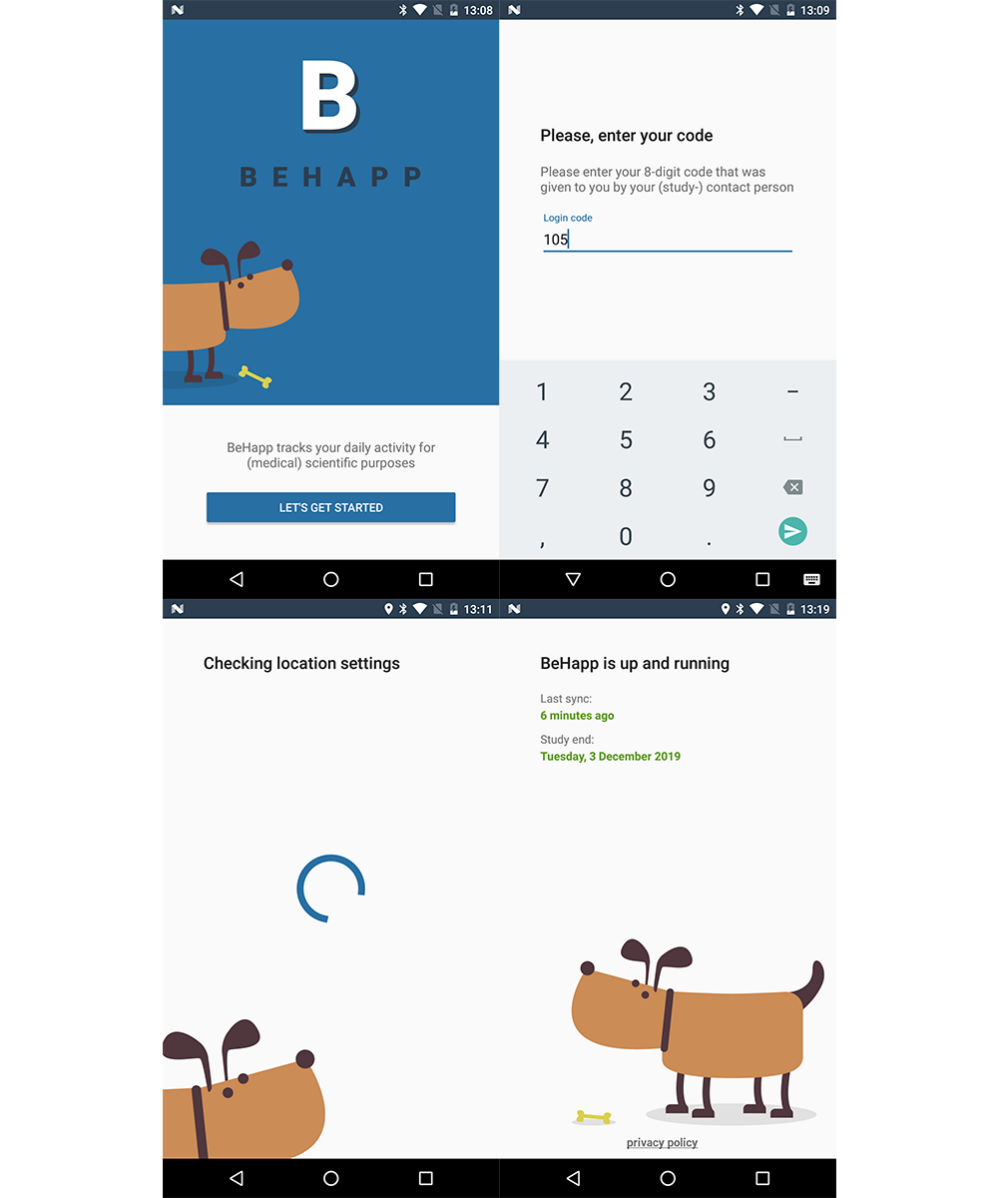
Screenshots of the installation and activation flow of the BEHAPP smartphone app.

This research is part of the BEHAPP project [13]. BEHAPP is a research platform that features the following 2 components:

1. The front end (Figure 1): a mobile app originally conceptualized for mobile passive monitoring (MPM) of human subjects. As a subfield of digital phenotyping, MPM refers to the practice of naturalistic observation through personal mobile devices exclusively relying on the collection of data that do not require any active input from the participant (an example of active input would be queries probing for emotions or situations such as in the experience sampling methods [14]).
2. The backend, which is the focus of this study: the backend is designed following the software as a service paradigm supporting multicenter studies for international research consortia, academic institutes, and the pharmaceutical industry. The work presented stems directly from our experience in accommodating the needs of industry partners and research groups representing large-scale study cohorts.

BEHAPP is comparable with initiatives such as MindLamp (Division of Digital Psychiatry, Beth Israel Deaconess Medical Center) [11], Beiwe (Onnela Laboratory, Harvard T H Chan School of Public Health) [15], Funf Open Sensing Framework [16], and the AWARE Framework [17]. All these platforms have in common that they collect various data streams through individual smartphones with the purpose of facilitating the study of human behavior in real time and in a natural (real-world) setting. BEHAPP also draws from a wide array of data collection sources that can be tailored to the needs of specific studies.

**Figure 2:**
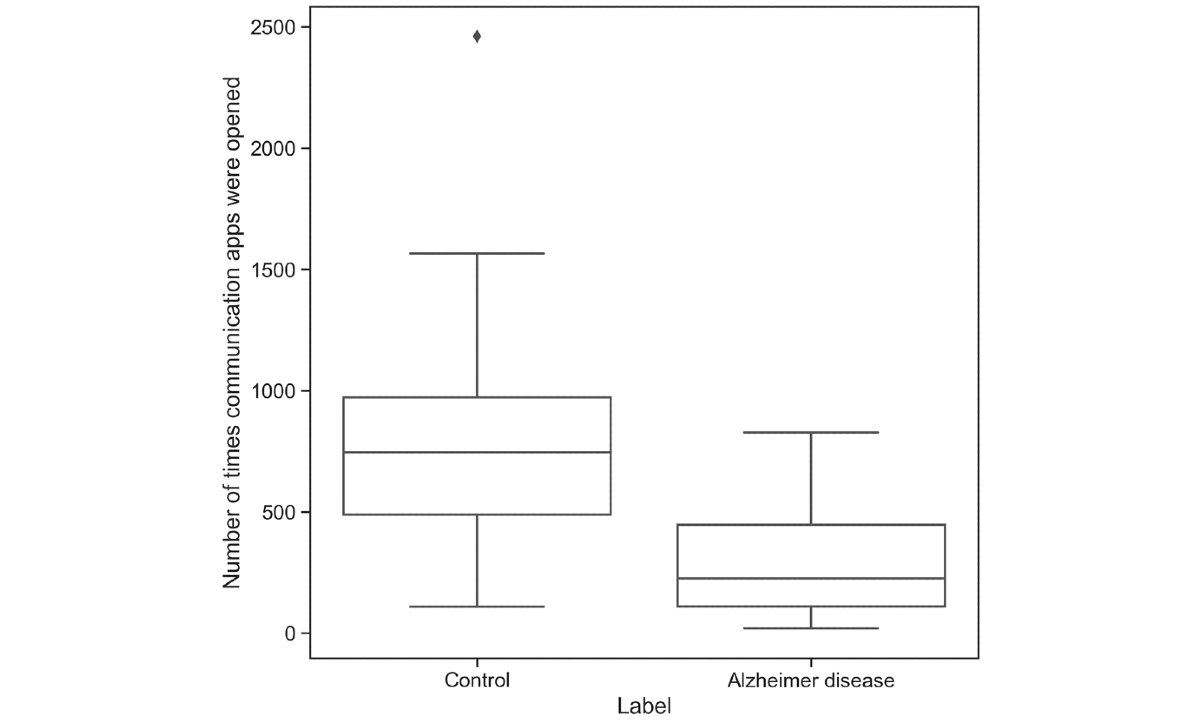
A group comparison data example on communication app use between Alzheimer disease patients and age-matched healthy controls (n=30) demonstrating the ability to observe differences in behavior through the use of BEHAPP’s behavioral monitoring platform. We measured a statistically significant difference in the number of times that communication apps were opened (median 746.5 and 226.0 times communication apps were opened; *P*=.003). The median age was 66.5 years (60% male and 40% female).

From the backend perspective, however, BEHAPP is different from the aforementioned initiatives. The alternatives require research teams to set up, manage, monitor, and secure the basic technical infrastructure themselves. Rather, BEHAPP is designed to be offered as a fully managed service aimed at low effort integration in (existing) studies. BEHAPP is currently used as an exploratory research instrument in general behavioral and clinical intervention studies. From these studies, we have provided a proof of principle demonstrating that these tools, the resulting data, and the clinical measures that we extract can be used to distinguish between neuropsychiatric patient and control groups [18], courtesy of the Psychiatric Ratings Using Intermediate Stratified Markers (PRISM) program [19]. For example, as depicted in Figure 2, we highlight a feature of communication app use. In this example, we observe that the overall frequency of communication app use is lower for the group with Alzheimer disease when compared with age-matched healthy controls. The service is currently exclusively used as a research instrument and is not employed as a tool to assist in the diagnosis, prevention, monitoring, treatment, or alleviation of disease, that is, at this moment in time, BEHAPP is not a medical device [20].

## Methods

### Overview

We focus on security and sustainability as a starting point for the development and operation of a behavioral monitoring platform. Within the concept of security, we address measures such as data isolation and encryption and highlight the importance of organizational security. We continue to discuss sustainability, explaining what is required to ensure that our platforms remain in service in a secure and stable way.

### Security

Security refers to the “absence of unauthorized access to information systems” [21]. Given the sensitivity of the data collected, the risk of (accidental) data leaks is a key consideration in setting up the platform. The current state of affairs on information security teaches us to step back and reflect critically on the design of our systems [22]. Regulatory frameworks such as the European General Data Protection Regulation (GDPR), in effect since 2018, must also be considered, given their important impact on technology development [23]. The GDPR expects initiatives involved with personal data to carry out a data protection impact assessment (DPIA) [24]. In a DPIA, we evaluate data sensitivity and how these data must be handled to prevent unauthorized access to and loss of personal data. Formulating a defense in-depth strategy is one such approach to address these concerns [25]. Defense in-depth strategies aim to ensure security by layering security measures at both the technical and organizational levels.

### Data Isolation

**Figure 3:**
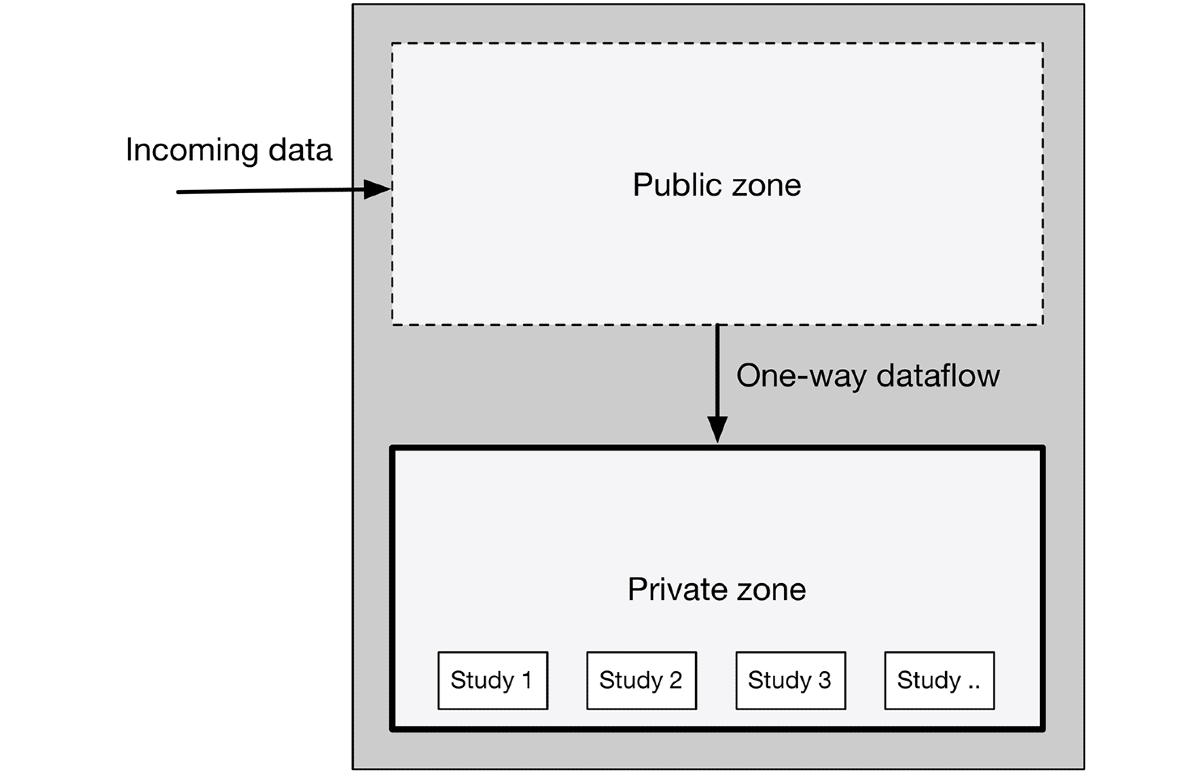
Public and private zone isolation overview featuring separated data storage.

Data isolation aims to minimize the exposure of data to data consumers. Data consumers can be both technical elements, such as (web) servers, and researchers interacting with data. The intent is to keep high-risk technical elements away from sensitive data. Technical elements connected to the public internet are particularly high risk because anyone can interact with and potentially exploit possible vulnerabilities of these elements. The most threatening are zero-day exploits, which are novel vulnerabilities that, by definition, are unpublished and thus difficult to protect against [26]. To address these risks, isolation of all sensitive data from publicly exposed technical elements is essential. This was achieved through network segmentation [27]*.* For BEHAPP, we established a public and private zone of operation, which are strictly separated (Figure 3). The public zone, which carries a higher risk profile, is responsible for receiving participant data from the outside world and immediately transmitting these data to the private zone. The private zone, which has limited outside world connectivity, receives and permanently stores participant data.

Furthermore, we defined a set of rules specifying the flow of data within the BEHAPP platform:

- Participant data may not be stored in a publicly exposed zone: The platform *needs* access to the public internet to receive data from smartphones and wearables. However, as explained, exposure to public internet access carries the risk of (part of) the platform (unknowingly) becoming compromised. Therefore, as a rule, any technical element that lives in BEHAPP’s public zone may not (temporarily) store any sensitive participant data, regardless of its encrypted state.
- Participant data may only flow in one direction, from the public to the private zone: Publicly exposed elements may only serve as an ingestion point for sensitive data to flow through to isolated private zones. Technical elements in BEHAPP’s public zone do not have any capability to interact with or retrieve participant data from the private zone and are completely unaware of the existence of these data.
- Participant data must be stored in a separate database for each study: The principle of least privileges dictates that automated operations and researchers only need to be assigned the absolute minimal set of authorizations to perform their task [28]. Therefore, any data collected by BEHAPP is segmented into separate data sets for each study, thereby further isolating the data allowing for granular access permissions. By separating study databases, our researchers can be authorized to access only specific studies. Furthermore, researchers are only given permission to *read* the data, which guarantees data integrity. This limits the potential fallout from data being lost or corrupted because of accidental leaks or compromised user accounts.

Note that data isolation by itself does not necessarily limit the processing of data. A private zone of operation can feature technical elements that perform automated data analysis tasks such as data enrichment and annotation, extraction of clinical endpoints, and data compliance and quality control checks. The results can then be written back to the public zone to enable direct reporting to study partners on the condition that the resulting information is fully anonymized. Finally, the resulting information can also be used for signaling purposes, for example, to address potential data quality and compliance issues that are found during automated data analysis runs. At BEHAPP, we are currently in the process of developing such capabilities.

### Encryption

Data encryption is the practice of obfuscating data by “converting information from an intelligible form into an unintelligible form” [29], thus rendering data unusable in case of a data leak. *The main challenge lies not in the application of encryption itself but in adequately managing encryption keys.* Protecting encryption keys is equally as important as protecting raw data; otherwise, the added level of protection will be de facto limited or nonexistent. A layered approach allows for responsible key and, subsequently, data management.

For BEHAPP, we employ a combination of symmetric and asymmetric encryption techniques to establish a closed encryption hierarchy. The main difference between both encryption techniques is in the key material used for the encryption and decryption of data. Symmetric encryption uses one key that is used for both encryption and decryption purposes. Asymmetric encryption uses 2 keys (a key pair), consisting of a public key and a private key. The public key is meant to encrypt data and therefore can be freely shared. The private key is meant for decrypting data and therefore needs to be stored safely [30].

The aforementioned encryption hierarchy consists of 2 levels:

1. Each study participant on the platform is assigned an asymmetric key pair. The key pair is generated in a private zone to prevent leakage of private key information. This key pair is used for encryption and decryption operations on sensitive participant data, such as the raw data that are collected.
2. Each private key is then encrypted itself before being stored in a central database. Private keys are encrypted using a key management service (KMS) [31] using symmetric encryption. A core characteristic of KMSs is that the key material itself never leaves the service. Instead, cryptographic operations are performed based on user authorization. This closes the encryption hierarchy because (unauthorized) database access by itself is not sufficient to decrypt data. Instead, access to both the database and KMS is required. For BEHAPP, we provide study-specific master keys within our KMS, segmenting the encryption hierarchy. The concept of isolation applies here as well, resulting in limited access to cryptographic operations on a per-study basis.

**Figure 4:**
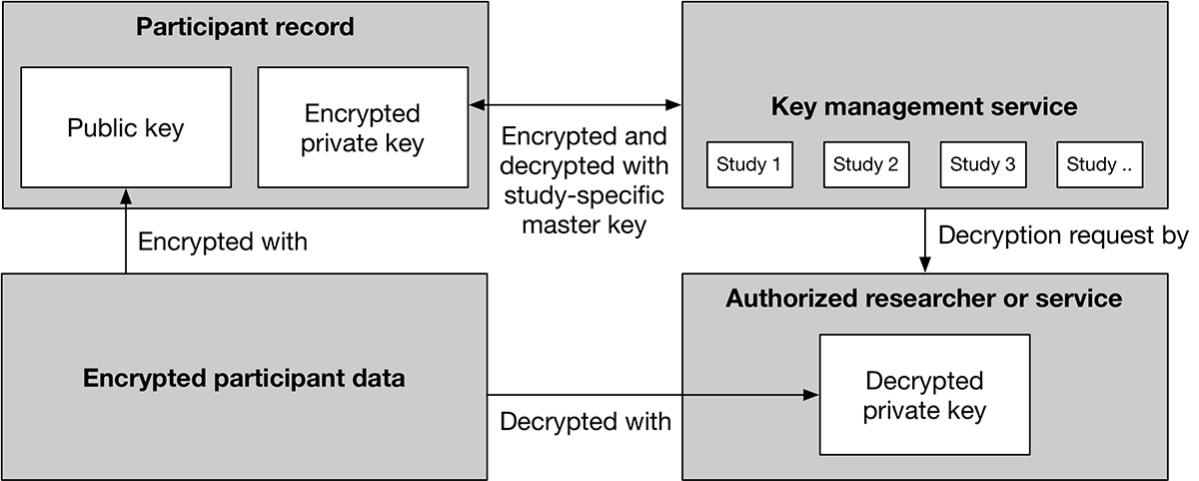
Three-step encryption model: (1) load a participant-bound encrypted private key, (2) request a decrypted version of the private key from the key management service, and (3) load participant data and decrypt with the decrypted private key.

This results in a 3-step encryption model, where (1) the researcher first loads the encrypted private key for a specific participant, (2) the researcher requests a decrypted version of this private key from the KMS, and (3) the researcher loads encrypted participant data from the database and decrypts the data using the decrypted private key obtained from the KMS (Figure 4). Authorizations such as data access permissions and permissions for cryptographic operations are checked at all stages of this process.

Finally, we adhere to the following rule on the use of encryption on the platform: data may only be collected, transported, and stored in an encrypted state. Encryption measures are continuously required to remain in place at all stages of the data lifecycle. In other words, data are only decrypted when necessary and discarded immediately after use. This means that data must be encrypted directly when they are collected through a smartphone or wearable device. For BEHAPP, we achieved this goal relying on the use of asymmetric encryption, as discussed earlier. We send a participant’s public key, which is safe to share, to their privately owned devices to be used by our app to encrypt any data that are collected. The encrypted data then remain in this state until they arrive at their destination in the private zone. Analytical workloads naturally require a decrypted view of the data. They are performed by keeping sensitive data in volatile memory only for the purpose of exploration, experimentation, and extraction of outcome measures. The data are then purged from memory.

### Information Security Culture

Technical measures alone are not sufficient to protect sensitive data collected by behavioral monitoring platforms. Users of information systems, such as researchers, play an important role in the secure use of information systems and, subsequently, any data under study. Raising awareness about security issues and safe practices is of paramount importance. Indeed, security incidents as a consequence of lack of awareness or negligence are common occurrences [32]. Researchers and newly entering Doctor of Philosophy (PhD) students, in particular, represent a class of users who, by the nature of their work, require access to sensitive data but who may not have any prior experience in the safe handling of these data. The challenge is to balance between maintaining security while not inhibiting (experimental) workflows of researchers [33]. We recommend the following guidelines to promote information security: (1) provide training and messaging to raise general security awareness and (2) provide seamless security to end users only minimally confronting them with security and compliance decisions [34].

First, raise awareness by establishing an information security policy (ISP) and ensure that all research staff are given an information security briefing. This briefing can be part of a general onboarding process, and its contents must be based on the ISP. An ISP is a document that defines “the rights and responsibilities of information resource users” [35]. The aim of this document is to explain how users responsibly handle sensitive data on a daily basis as part of their work. An effective document is understandable, practical, and inclusive of the needs of researchers. The ISP of the BEHAPP project is targeted at an audience with above-average computer literacy (PhD students, postdocs, and principal investigators). Given this audience and the sensitive data that we use, we specify concrete rules for high-risk areas of attention, such as data flow. For example, we concretely specify that data may only reside on the central server and that local copies on individuals’ devices may temporarily exist for analytical purposes only. We also clearly specify that the data may not be uploaded or transferred to any other service or device (eg, for data enrichment purposes) without discussing the purpose and scope of this intention with the team.

Second, seamless security is provided through software development effort. We recommend investing in the development of custom toolkits that are responsible for the heavy lifting around safely loading, decrypting, exploring, and analyzing data. Furthermore, user authentication should be based on multifactor authentication strategies adhering to modern password security guidelines, as defined in National Institute of Standards and Technology 800-63-3 [36]. This considerably eases the burden of security compliance–conscious behavior. At BEHAPP, we developed an internal tool based on these exact principles, called the behapp-data-kit. The kit is a Python package or library aimed at ease of use and security, offering a simple programming interface for data exploration and analysis. Meanwhile, security compliance is handled hidden from the researchers’ perspective. For example, the data kit automatically manages local copies of data, ensuring that these copies are encrypted and removed when they are older than 14 days. Furthermore, when loading the data into active working memory for analysis, the kit ensures that the decryption keys necessary for decrypting the data are only held in memory for the shortest amount of time, explicitly deleting these keys when they are no longer required. Finally, the kit relies on a single multifactor authentication strategy, which results in a high level of trust without researchers having to deal with multiple sets of credentials.

Third, schedule and hold weekly team meetings to discuss any (potential) use of sensitive data. Reflect on whether the use complies with the ISP and if the ISP still holds relevance or if an adjustment is required. Be especially mindful of *shadow security.* Shadow security refers to ad hoc practices devised by security-conscious employees that are not compliant with formally prescribed security policies in an effort to achieve a more optimal balance between getting work done and protecting information security [37]. Kirlappos et al [37] recommend learning from these practices, arguing that without engaging with users on these practices, one cannot claim that a specified security infrastructure exists as intended. For example, at BEHAPP, over time, the security policy proved to be difficult to accommodate to researchers who were not directly affiliated with the team, such as graduate students working on temporary assignments. The logistics of account and hardware security key provisioning did not fit the short and temporary character of these projects. Thus, the following shadow security practice emerged: manual data exports were generated for graduate students, but these exports were limited to fully anonymized clinical measure overviews. Although initially any form of manual data exports was formally prohibited, the adjustment of providing anonymized exports offered a workable middle ground and has been adopted as a standard practice.

### Sustainability

Sustainability refers to the ability to ensure availability, support, and improvement of the software products and services that we create [38]. We highlight 3 qualities that are closely connected to the concept of sustainability: maintainability, reliability, and scalability [5].

Maintainability is the degree of effectiveness at which software products can be modified [39-41], which depends on multiple factors such as documentation, design, and the consistent application of clean coding standards. Maintenance is an essential part of operating a software service. *The foundations that we built upon, such as mobile operating systems and web application frameworks, change continuously and thus require frequent modification of our own code.* Failure to do so results in diminished service performance, possible loss of functionality, and increased exposure to security threats. Maintenance is especially relevant with regard to mobile apps that we employ as our measurement instruments. Each yearly upgrade of mobile platforms brings about changes that often directly impact the quality of the data that we intend to collect. In addition, noncompliance with continuously changing Apple’s App Store and Google’s Play Store policies may result in apps being removed altogether. Thus, keeping up with maintenance-related tasks is essential in that it avoids or at the very least mitigates the potential negative impact of platform upgrades and policy changes. Unfortunately, negative changes cannot always be avoided. For example, over the current 4-year development span with BEHAPP, we have experienced many changes in Google’s terms of service for the distribution of our Android (Google) app through the Play Store. One change, in particular, revolved around Google’s strides to curb malicious apps invading the privacy of their users. This change, which came into effect in March 2019, limited access to call and text messaging logs for the majority of apps distributed through Google’s Play Store [42]. This change was unfortunate, as call logs are very expressive of the communicative behavior of participants. Fortunately, we could work around this problem by directly distributing our app to our end users and thus circumvent the Play Store through sideloading.

Reliability refers to the probability of failure-free operation of a software product [43]. The goal is to ensure that our platforms are highly available, keeping service downtime at a minimum. However, it must be noted that experimental research initiatives may typically have wider tolerances for service uptime requirements. This changes when our behavioral monitoring platforms evolve to a stage where they are involved with mission critical workloads, for example, when their continuity is essential to large-scale research endeavors or when they provide important information to clinical care processes.

Scalability is the ability of a software product to adapt to changing circumstances in demand [44]. The rapid adoption of behavioral monitoring platforms and increasing cohort sizes require us to ensure that our software products can sustain the stresses of increases in demand.

Finally, note that the aforementioned quality attributes and the corresponding responsibilities not only apply to the programs and code behind our platforms but also to every supporting information technology (IT) infrastructure element that is required to bring our platform into service. This can span the full range of elements such as networking, storage, physical servers, virtualization, operating systems, and web server software. All these elements require setup and maintenance for stable and continued secure operation. The proverb that a chain is only as strong as its weakest link applies here: a vulnerability or weakness in any supporting IT element will affect the other elements as well, including our behavioral monitoring platforms. *Thus, we are not only required to develop a secure and sustainable behavioral monitoring platform but we also need to ensure that the supporting IT infrastructure is equally secure and sustainable.* Unfortunately, both the knowledge and resources required to do so are extensive and form a barrier to entry.

At BEHAPP, we have built a service relying on fully managed IT infrastructure components offered by large cloud providers, focusing in particular on an upcoming class of products known as serverless computing. With serverless computing, the cloud provider is responsible for maintaining and securing the majority of the required IT infrastructure, leaving us to focus on our platform only [45]. This includes the ability to (rapidly) scale, which means that we can flexibly meet any changes in demand.

## Results

**Figure 5:**
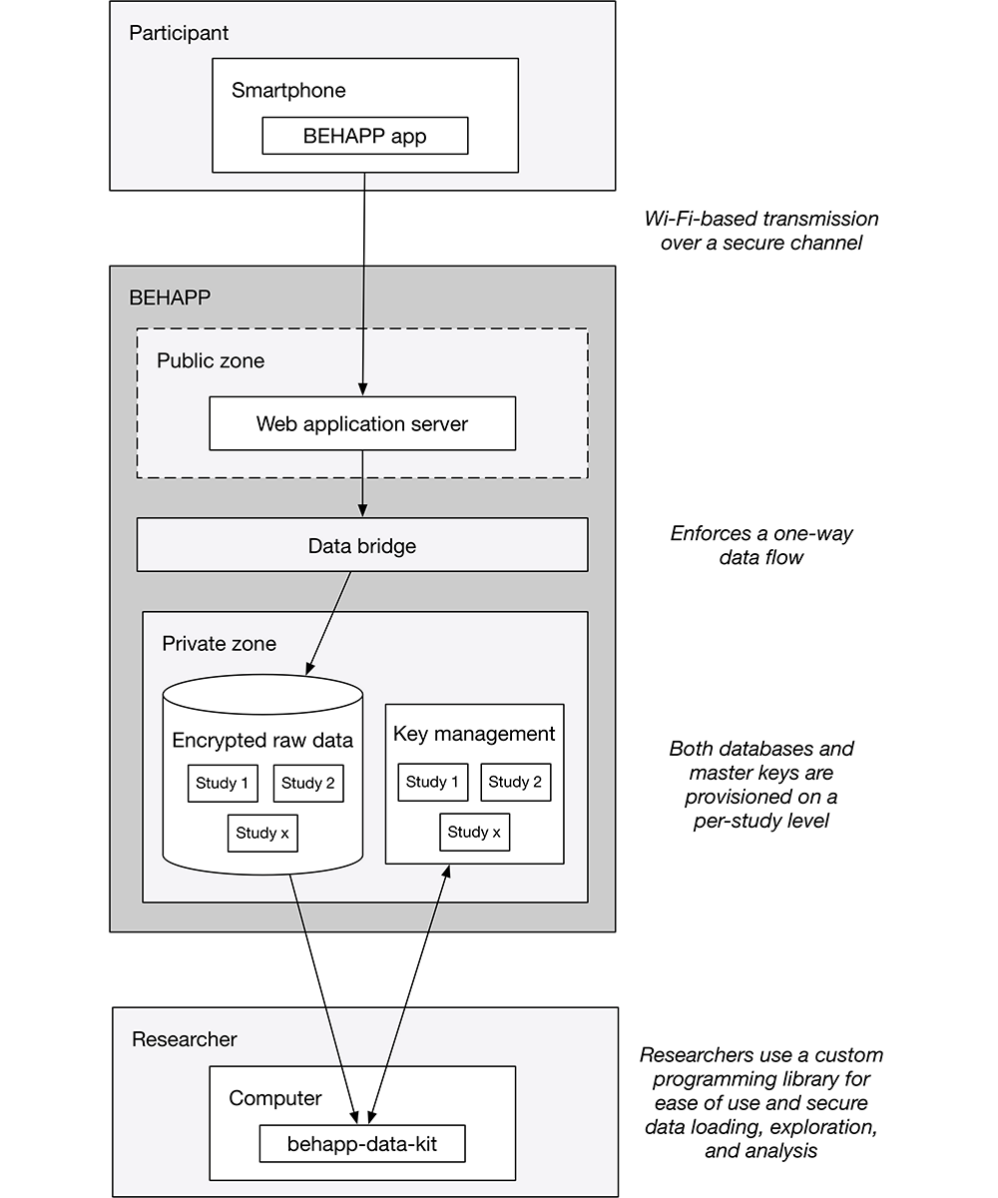
BEHAPP data flow for the collection and analysis of data using smartphone-based participant data.

Here, we introduce the base BEHAPP data flow for the collection and analysis of raw data (Figure 5). The flow implements the requirements and guidelines, as discussed in the aforementioned sections:

1. Data collection starts at the personal devices of participants that run the BEHAPP smartphone app. The app unobtrusively collects various types of data descriptive of the participants’ behavior. Any data collected were encrypted immediately and temporarily stored on the device.
2. The data are sent to the public facing the web application server. The smartphone app is programmed to upload these data on a fixed interval and attempts to do so on the condition that a Wi-Fi (or unmetered network) is available. Given that some modes of data collection can yield substantial amounts of data, we do not want to risk accidentally consuming the data plans of our participants. Data transmission always occurs over a secured connection. Once received, the web application server immediately passes the data on to the data bridge.
3. The *data bridge* is not a formal concept but represents a technical construct responsible for enforcing a one-way data flow, which can be achieved in multiple ways. BEHAPP employs message queues configured with minimized permissions to realize unidirectional flow of data.
4. The private zone receives data from the bridge and is responsible for placing the raw data in the corresponding study database. In this way, researchers can access only a select number of databases instead of being able to access the full data set.
5. Finally, researchers use a custom programming library, the behapp-data-kit, designed for ease of use and security. The kit supports data loading, exploration, and analysis and is aimed at security and ease of use. Security compliance, such as maintaining data encryption and enforcing short-lived life cycles of decrypted and locally stored data, is hidden from view. This allows researchers to focus on the task of data analysis while maintaining a secure level of use.

## Discussion

### Principal Findings

We present a data flow design that includes a set of requirements and operational guidelines for the realization and responsible operation of a digital behavioral monitoring platform. Having done so, we recognize the following limitations in the proposed work.

First, the proposed data isolation measures prevent the public elements of the platform from accessing (raw) sensitive data. In other words, by design, researchers will not be able to retrieve sensitive data in an automated way. Instead, data are provided (both raw and extracted features) manually on request. This may not be an alluring policy, as it implies that researchers cannot have immediate data access. However, given the risk profile of publicly connected technologies, we opted to go for a risk-averse approach and stand by the decision to strictly separate sensitive data to protect the participants under observation by the platform. In line with the aforementioned data isolation rules, once received, any form of sensitive data may not leave the service through a publicly connected element. However, there are ways to automate the retrieval of sensitive data, for example, through the use of secure file transfer mechanisms initiated in private zones, which should be the focus of further development and future studies.

Second, by implementing both technical and organizational measures to protect the data of our participants, we realize that there is room for improvement on the data protection front by the application of anonymization methods. BEHAPP’s data, while fully encrypted, are not fully anonymous and have, considering the location data that are collected from, the potential for direct identification of individuals. However, although strategies exist for anonymizing location data, its effectiveness is still under debate [46]. As a consequence, analytics endeavoring down the line could be limited by the application of anonymization strategies. For example, we would lose the ability to retrospectively annotate locations by adding meaning to specific points (eg, schools, hospitals, and sports). However, note that although raw location data are considered as identifiable information, the behavioral features that we extract and subsequently report are not. Features such as *total time spent at home* and *total number of unique places visited* are highly interpretable and expressive of one’s mobility without having to refer to any geographical type of data and as such can reside in publicly connected zones.

Third, considering our aforementioned proof of principle, we demonstrate the capability to accurately distinguish between patient and control groups. We feel it is time to look ahead and ensure that the models that we intend to build, be it for predictive or classification purposes, are not only properly validated but also compliant and sustainable from an ethical perspective. Many unintuitive and unverifiable inferences can be drawn from personal data, which can potentially result in negative consequences for the individuals for whom the inferences are drawn. Although the GDPR demands model transparency, the subject of inferential analytics is not well regulated [47]. Given our medical scientific operating context, we should tread carefully and actively work toward the creation of transparent models with limited application scopes to avoid negatively affecting our subjects under study.

Fourth, despite our efforts to simplify the design and minimize the operational overhead of running a behavioral monitoring platform, we realize that the level of complexity and the demands imposed on a research team may still leave this type of instrumentation out of reach for many independently operating research groups. However, we strongly feel that this is the minimum standard for responsibly operating such a platform. Self-hosted open-source models may be vulnerable in this regard and, therefore, are not the most secure and sustainable way forward. As argued in this paper, the responsible operation of such platforms extends beyond installing a client and server application, and we have to consider the underlying IT infrastructure as well. In our experience, the broad level of responsibilities tied to operating a behavioral monitoring platform warrants the inclusion of a dedicated team responsible for development, maintenance, monitoring, and security tasks. A more workable model would be to concentrate the required operational effort in a limited number of initiatives capable of supporting multiple studies. With BEHAPP, we aim to be one of these initiatives.

However, the open-source model is not without merit. Indeed, Torous et al [1,10] regularly raise a valid and important point about the lack of interoperability and interpretability of results across the whole spectrum of digital phenotyping initiatives. The open-source model and, consequently, the free distribution of these platforms is meant to address that problem by putting the technology in the hands of many, thereby ultimately contributing to a more uniform approach toward data collection and analysis. However, we think that the solution to interoperability and comparability does not rely on a single developer, mainly because the underlying problem is an overall lack of transparency in the methodology and specifications descriptive of data collection and data processing flows. Importantly, we need scientific reports to be accompanied with metadata descriptive of the format of data (attributes, shape, size, and semantics) at every stage of collection and analysis, starting from raw data to the clinical measures that we extract. These aspects, just as security and sustainability, are largely overlooked in most scientific reports. With phenotypic outcomes currently taking center stage, we unfortunately limit ourselves in building collective knowledge toward enabling reproducible science.

### Conclusions

In search of strategies for secure and sustainable digital phenotyping, we identified a gap in the available knowledge related to the establishment of secure and sustainable platforms that drive such research initiatives. Here, we address it by providing a foundation including requirements and operational guidelines focusing on key elements such as the application of encryption, data isolation, and organizational security culture. Members of ethical research boards should consider using the security principles outlined in this manuscript in their evaluation of the privacy of study participants in research proposals. Principal investigators should account for these essential components while budgeting their grant proposals, keeping in mind that security and maintenance must be adequately addressed in any research plan that includes the use of a digital phenotyping platform. Taken together, this work contributes to the foundations on which digital phenotyping strategies can be operated in a safe and sustainable way, allowing for the collection of real-time, quantitative, and longitudinal behavioral data that are expected to generate novel insights and possibly support concrete innovations in clinical care.

## Abbreviations

DPIA: Data Protection Impact Assessment
GDPR: General Data Protection Regulation
ISP: Information Security Policy
IT: information technology
KMS: Key Management Service
MPM: Mobile Passive Monitoring
